# Mental Health and Autosomal Dominant Polycystic Kidney Disease: A Narrative Review

**DOI:** 10.34067/KID.0000000000000504

**Published:** 2024-07-08

**Authors:** Niloufar Ebrahimi, Pranav S. Garimella, Fouad T. Chebib, Matthew A. Sparks, Edgar V. Lerma, Mohadese Golsorkhi, Zohreh Gholizadeh Ghozloujeh, Amir Abdipour, Sayna Norouzi

**Affiliations:** 1 Division of Nephrology, Department of Medicine, Loma Linda University Medical Center, Loma Linda, California; 2 Division of Nephrology and Hypertension, Department of Medicine, University of California–San Diego, San Diego, California; 3 Division of Nephrology and Hypertension, Mayo Clinic, Jacksonville, Florida; 4 Department of Medicine, Division of Nephrology, Duke University Medical Center, Durham, North Carolina; 5 Section of Nephrology, Department of Medicine, University of Illinois at Chicago, Chicago, Illinois

**Keywords:** ADPKD, genetic renal disease

## Abstract

Autosomal dominant polycystic kidney disease (ADPKD) is a genetic disorder marked by the development of cysts in the kidneys and other organs, leading to diverse clinical manifestations, including kidney failure. The psychological burden of ADPKD is substantial, with significant contributors including pain, daily life disruptions, depression, anxiety, and the guilt associated with transmitting ADPKD to offspring. This review details the psychological impacts of ADPKD on patients, addressing how they navigate physical and emotional challenges, including pain management, genetic guilt, mood disorders, and disease acceptance. This review also underscores the need for comprehensive research into the psychological aspects of ADPKD, focusing on the prevalence and contributing factors of emotional distress and identifying effective strategies for managing anxiety and depression. Furthermore, it highlights the importance of understanding the diverse factors that influence patients' quality of life and advocates for holistic interventions to address these psychological challenges.

## Background

**Figure 1:**
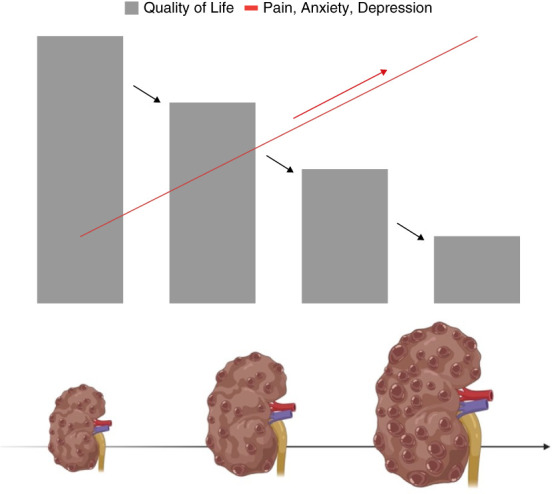
**This figure illustrates the progressive disease burden of ADPKD over time.** As the kidneys enlarge and renal function diminishes, patients experience a decline in QoL accompanied by increases in pain, anxiety, and depression. The figure emphasizes the inverse relationship between TKV and both physical and mental well-being, underscoring the impact of ADPKD progression on patients' health. ADPKD, autosomal dominant polycystic kidney disease; QoL, quality of life; TKV, total kidney volume.

Autosomal dominant polycystic kidney disease (ADPKD) is the most prevalent hereditary kidney disorder; it affects 1 in 1000 live births in the United States.^1^ Diagnosis is often made in a clinical setting in an asymptomatic patient with a positive family history of ADPKD or incidental finding of imaging because of other medical conditions.^1^ The clinical presentation of ADPKD is highly variable.^2,3^ While many patients remain asymptomatic and might be diagnosed incidentally during abdominal imaging for other indications, others might experience frequent back or flank pain due to cyst hemorrhage, cyst infection, nephrolithiasis, or chronic pain related to enlarged kidneys.^2^ Studies have reported that pain is an important but often overlooked symptom in patients with ADPKD and can contribute to a lower quality of life (QoL) and a high risk of experiencing depression and anxiety.^4,5^ Even patients with asymptomatic or early-stage ADPKD reported lower life satisfaction and more suppressed emotions compared with healthy individuals.^6^ Patients without severe symptoms often struggle to accept and understand the significance and implications of their ADPKD diagnosis.^7^ In some cases, younger individuals demonstrated resentment toward their parents for passing on ADPKD, feeling unprepared for the unexpected diagnosis, and unaware of the disease's genetic transmission potential.^8^ This burden may manifest as persistent genetic guilt, creating a sense of self-blame and responsibility for transmitting the condition to future generations.^7,9^ Despite the well-documented physical aspects, there is a notable scarcity of comprehensive studies on the QoL, psychosocial impact, and overall experience of living with ADPKD, particularly in the early stages of the disease.^10^ Given the role of mental well-being in the physical health of patients and treatment success, this review aims to highlight the psychological impacts induced by ADPKD, including anxiety, depression, pain, genetic guilt, and emotional suppression. As the progression of ADPKD advances, there is decline in QoL, accompanied by increases in pain, anxiety, and depression (Figure 1).

## Anxiety, Depression, and Uncertainty Related to Disease Progression

Many patients with ADPKD report experiencing depression, helplessness, and anxiety about their disease progression to ESKD. In the early stages of the ADPKD, emotional disturbances primarily manifest in three areas: (*1*) loss and concerns related to life expectancy, overall health, and the ability to engage in valued activities; (*2*) uncertainty, characterized by frustration and anxiety about the unpredictable progression of the disease; and (*3*) fear and worry for both themselves or their children about the potential course and outcomes of the disease.^10^ Barros *et al.* evaluated anxiety, depression, and QoL in 38 patients with ADPKD. The study revealed that 37.1% experienced mild state anxiety, a temporary and situational form of anxiety, while 47.4% exhibited moderate trait anxiety, a predisposition to experience anxiety across various situations and over time.^11^ There was no correlation between laboratory findings and anxiety levels. Higher anxiety levels were observed in women, single individuals, and those with lower educational attainment. Depression was reported in 60.5% of patients with ADPKD, yet none of the patients were prescribed antidepressants or receiving therapy for depression. The most common symptoms reported in the Beck depression inventory included loss of libido, sleep disturbance, fatigue, and difficulty working.^11,12^ A cross-sectional study on 100 patients with ADPKD using the three-level EuroQol five-dimension three-level questionnaire, which measures mobility, self-care, usual activities, pain/discomfort, and anxiety/depression, found that up to 30% of patients experienced extreme anxiety and depression. There was also a statistically significant increase in anxiety levels as the disease progressed.^13^

**Figure 2:**
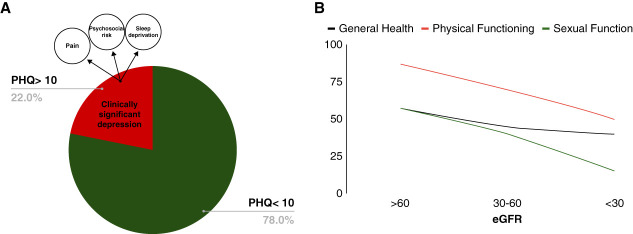
**The prevalence of depression among patients with ADPKD.** (A) Pie chart illustrating the prevalence of clinically significant depression (PHQ >10) among patients with ADPKD), accounting for 22% of the population based on the study of Simms *et al.*^14^ The chart highlights key factors contributing to depression, including pain, sleep deprivation, and psychological risk. (B) Graph demonstrating the decline in general health, physical functioning, and sexual function in relation to eGFR and CKD stages. The graph shows a downward trend in all three parameters as eGFR decreases, based on the study of Simms *et al.*^14^ PHQ, patient health questionnaire.

Studies have shown that patients with ADPKD suppress their negative emotions, such as anxiety and depression, more intensely than their healthy peers.^13^ A significant and direct relationship between extreme levels of anxiety/depression and degree of CKD severity was found (*P* < 0.001). In addition, patients with ADPKD who have more depressive complaints have less dietary compliance.^13^ This suggests that early detection and treatment of psychological difficulties may affect the course of ADPKD and may be as important as the medical treatment.^13^ Simms *et al.* evaluated depression among 158 patients with ADPKD using a patient health questionnaire-9 (PHQ-9) questionnaire. They found that 22% of patients reported clinically significant depression (PHQ-9 >10). Depressed mood in patients with ADPKD was associated with pain, sleep deprivation, and increased psychosocial risk. In addition, lower eGFR, female sex, and larger kidney size were associated with higher levels of depression, poorer QoL, and overall adverse psychosocial well-being (Figure 2).^11,14^ Baker *et al.* conducted a study involving 80 patients with ADPKD with CKD stages 1–3.^10^ Among the 25 patients diagnosed with CKD stage 1 or 2, 22 (88%) reported symptoms, such as back abdominal or kidney pain, fatigue, breathlessness, weakness, and general malaise. Notably, 56% reported feelings of hopelessness and helplessness. They described frustration and depression caused by the uncertainty related to the progression of their disease.^10^

## Pain Contributing to Emotional Distress

Pain, a prevalent symptom in patients with ADPKD, significantly diminishes QoL. Effective management requires a combination of pharmacological and nonpharmacological approaches.^15^ In severe cases, surgical or radiological interventions such as cyst aspiration and sclerotherapy, cyst fenestration, or partial hepatectomy may be necessary because of the debilitating impact on QoL.^16,17^ The burden of symptoms in ADPKD, including pain, hypertension, and complications arising from kidney enlargement, plays a substantial role in escalating psychological distress (Table 1).

**Table 1:** Summary of some of the included studies in this review

Study	Summary of the Findings
Barros *et al.*^11^ (2011)	• Depression was found in 60.5% of patients diagnosed with ADPKD • Anxiety and depression were more prevalent among patients with lower educational levels • QoL was worse among women, unmarried individuals, White patients, and those with lower educational level • Patients exhibited elevated risks of sleep disturbances and suicide • Lower economic status, limited access to kidney disease prevention programs, and higher prevalence of malnutrition and depression were correlated
Baker *et al.*^10^ (2015)	• Patients described a sense of loss regarding having children, life expectancy, living a healthy life, and valued activities; they expressed frustration and anxiety due to uncertainty about disease progression, often exacerbated by a lack of definitive information from their physician • Most patients described fear for themselves or their children concerning the course and outcome of the disease; this fear, often worsened by uncertainty, was associated with emotional distress, including anxiety and depression • Some patients experienced deep emotions due to witnessing the consequences of ADPKD in close relatives or the sudden and unexpected nature of their diagnosis
Simms *et al.*^14^ (2016)	• Female sex is identified as an independent risk factor of adverse psychosocial health • Advancing age is associated with reduced QoL • Larger kidney size is linked to heightened psychosocial risk • Depression is correlated with disrupted sleep and various adverse patient-reported outcomes • High rates of diminished QoL, depression, and heightened psychosocial risk are observed in a large group of ADPKD patients before renal replacement therapy
Jankowska *et al.*^6^ (2022)	• Asymptomatic patients with ADPKD exhibit greater suppression of three basic negative emotions: anger, anxiety, and depressed mood; this emotional suppression increases with age in these patients • Patients with ADPKD report lower life satisfaction compared with healthy controls • The overall degree of acceptance of illness in ADPKD is high, with satisfactory psychological adjustment observed in patients during the early stages of the disease
Winterbottom *et al.*^5^ (2022)	• Patients with ADPKD consistently report poorer general health, reduced energy, poorer physical, mental, and emotional health, and limitations in social functioning • Flank pain and moderate decline in eGFR contribute to reduced QoL in patients with ADPKD

ADPKD, autosomal dominant polycystic kidney disease; QoL, quality of life.

In the study by Tong *et al.*, some patients reported that their physicians did not recognize the severity of their pain and failed to take it seriously.^7^ Patients reported the need to convince their physicians about the severity of their pain, a phenomenon described as medical trivialization.^7^ Traditional assessment tools, such as EuroQol 5-dimension 3-level and kidney disease quality of life-short form version 1.3, may not adequately capture the psychological burden in ADPKD because of the unique complexities associated with pain and its impact on QoL, which are not common features of other forms of kidney disease. To address this gap, Oberdhan *et al.* developed the ADPKD Impact Scale, which contains 14 items across physical, emotional, and fatigue domains to evaluate the impact of ADPKD on patients' lives. In addition, the ADPKD Impact Scale includes four supplementary questions addressing guilt, sleep, abdomen size/shape, and urinary frequency/urgency, providing a comprehensive understanding of ADPKD patients' experiences.^18^ In a subsequent study, Oberdhan *et al.* introduced the ADPKD Pain and Discomfort Scale, specifically designed to capture pain-related aspects in patients with ADPKD.^19^ These validated tools offer a standardized approach for capturing patient-reported outcomes in ADPKD enhancing the understanding of the disease's impact.^18,19^ Given that pain significantly contributes to the mental and the physical burden aspects of ADPKD and leads to considerable decline in health-related QoL, these tools could be effectively used in clinical practice and clinical trials to address this aspect comprehensively.^20^

Physical discomfort and mental health are inextricably linked; a decline in one often worsens the other.^6^ A study involving 97 patients with ADPKD showed that pain is a common issue in their everyday lives, often underestimated by physicians.^21^ Patients experienced limitations in their daily activities because of unexplained and unpredictable pain and reported that pain management was suboptimal, with pain rarely discussed during health care appointments.^21^ Pain control in patients with CKD is challenging because of limitations on the use of nonsteroidal analgesics and opioids, given the concerns about worsening kidney function or side effects. Importantly, patients with ADPKD reported having limited choices in the management of their pain and preferred active participation in comprehensive conversations concerning their pain, including medications, nonpharmacological methods, and self-management strategies.^7^ In a Polycystic Kidney Disease (standardized outcomes in nephrology-polycystic kidney disease) Consensus Workshop consisting of 58 participants (11 patients/caregivers and 47 health professionals), patients expressed difficulty in effectively capturing pain to accurately determine its source and severity. Owing to frequent challenges in accurately identifying the cause or source of pain, the consensus workshop recommended including pain in general terms (rather than ADPKD pain). The participants also emphasized the importance of developing a standardized and validated patient-reported outcome measure to capture pain.^22^

## Genetic Guilt and Resentment

Most patients with ADPKD have a family history (up to 20% might have a negative or unknown family history of ADPKD). Approximately 78% and 15% of ADPKD cases are due to pathogenic variants in *PKD1* and *PKD2*, respectively. Truncating *PKD1* pathogenic variants are associated to a more severe form of the disease and an earlier onset of ESKD.^23^ Genetic guilt, stemming from the hereditary nature of ADPKD, contributes to anxiety and resentment, particularly among younger individuals. In a study of 138 patients with ADPKD, 62% reported feeling guilt about passing the disease to their children.^14^ This feeling intensifies when conception occurs before parental diagnosis, particularly in cases of *de novo* mutations.^24^ Such guilt can lead some patients to avoid having children and actively seek medical advice on parenthood; however, for others, the decision to have children remains a personal choice.^25^ Preimplantation genetic testing allows prospective parents to avoid passing on heritable diseases, including monogenic polycystic kidney disease, to their children. The preimplantation genetic testing process involves generating embryos through *in vitro* fertilization, testing these embryos, and selectively transferring those that do not carry the specific disease-causing variant. Despite technological advancements, many challenges remain, including ethical, regulatory, and financial considerations.^26^ Genetic counselors play a crucial role in helping patients with ADPKD understand their risk of passing the disease to their offspring. They are key team players in obtaining and interpreting genetic testing results. Counselors facilitate open family communication about genetic risks and provide coping strategies and resources tailored to individual needs.^27^ This support enables patients to make informed decisions regarding genetic testing, navigate interpretation of results including variants of uncertain significance, understand non–kidney-related aspects of hereditary kidney diseases, such as implications to life insurance, and receive education on genetic test results. In addition, counselors offer support for family planning, ultimately alleviating psychological burdens.^27^

## Disease Acceptance and Emotional Suppression

Disease acceptance and emotional suppression vary among individuals with ADPKD, ranging from a high level of disease acceptance in asymptomatic patients to pronounced emotional suppression in later stages.^6^ A 2022 study enrolled 50 healthy individuals and 50 asymptomatic patients with ADPKD (eGFR >60 ml/min per 1.73 m^2^) to investigate coping mechanisms and emotional suppression in the early stages of the disease.^6^ Patients completed the Acceptance of Illness Scale, Courtauld Emotional Control Scale, and Satisfaction with Life Scale questionnaires. The results indicated that patients with ADPKD suppressed their negative emotions, such as anxiety and depression, more intensely than their healthy peers (range 26–74, mean 49 [10] points in the ADPKD group and range 25–52, mean 38 [7] points in the healthy group; *t*=−6.04, *P* < 0.001), and this tendency toward emotional suppression was found to be intensified with age. In addition, satisfaction with life was significantly lower in the ADPKD group compared with healthy controls (range 8–34, mean 21 [5] versus range 16–34, mean 24 [4]; *t*=3.25, *P* = 0.002).^6^

## QoL

The QoL in patients with ADPKD is intricately linked to both physical and mental health and varies significantly across different stages of CKD. In a retrospective study by Eriksson *et al.*, patients with ADPKD without CKD (defined as a eGFR <60 ml/min per 1.73 m^2^) and those with CKD stage 3 have higher QoL than patients with CKD 4 and those with ESKD receiving dialysis.^28^ Notably, patients who underwent kidney transplantation reported higher QoL scores compared with those with CKD 4 and ESKD.^28^ In another study examining QoL in patients with ADPKD, 465 individuals with a preserved eGFR were asked to complete the kidney disease quality of life-short form version 1.3 questionnaire disease. The authors stratified patients on the basis of eGFR, total kidney volume (TKV), and polycystic kidney disease genotype, providing a comprehensive analysis of disease severity and its impact on QoL.^5^ Patients with lower eGFR were less likely to be engaged in full-time employment and reported lower sexual function. A significant negative association was found in the energy/vitality subscore, with QoL worsening as TKV increased.^5^ This study highlights a notable deterioration in QoL across various domains, challenging earlier beliefs that kidney volume affects QoL only beyond a certain threshold.^5^ These finding are echoed in the genetic psychosocial risk instrument tool, which suggests a correlation between larger kidneys, lower eGFR, and increased psychosocial risks. However, the short form 36, despite its widespread use, was unable to demonstrate clinically relevant dissatisfaction in QoL, particularly regarding the correlation between TKV and health-related QoL scores.^14,29,30^

## Conclusion

The diagnosis of ADPKD introduces significant uncertainty for the patients and their caregivers. This uncertainty stems from the disease's high phenotypic variability, the potential for complications beyond kidney failure, and the current lack of treatments that completely halt disease progression. The hereditary nature of ADPKD adds complexity, heightening concerns about the disease's impact on family members. This multifaceted uncertainty surrounding ADPKD not only complicates medical management but also poses psychological challenges, necessitating comprehensive strategies to support affected individuals and their families. The ambiguity regarding the disease trajectory particularly onset of kidney failure can be distressing for patients and their family members. This highlights the importance of robust support networks that address both the physical and emotional challenges posed by ADPKD.^10^ Interestingly, higher levels of education, which may correlate with better socioeconomic status and health care access, are associated with improved coping outcomes.^29^ This suggests that enhancing education and health care access could play a crucial role in mitigating the psychological impact of ADPKD on patients and their families.^11,29^ The assessment and management of anxiety and depression in patients with ADPKD require a multifaceted approach. Integrating regular psychological evaluations into routine care is crucial for early detection and timely intervention, thereby enhancing overall QoL. Effective management could include a combination of pharmacological treatments, counseling, cognitive behavioral therapy, and participation in support groups. Educating patients about ADPKD, including its nature, potential outcomes, and management strategies, can alleviate anxiety and depression. Empowering patients to actively participate in their care fosters a sense of control, which is vital in managing chronic diseases. Social support is important in mitigating anxiety and depression; a robust support system encompassing family, patient communities, and effective coping strategies can significantly diminish the psychological impacts of living with ADPKD.

Neglecting psychosocial issues has the potential to impose a socioeconomic burden on individuals, arising from choices in career and financial planning that may adversely affect them and their families over an extended period.^31^ ADPKD affects not only patients but also their family members, who are involved in caregiving and support. This involvement can disrupt sleep patterns, raise concerns about medical treatments, prompt reliance on cultural and religious beliefs, limit personal freedoms because of caregiving obligations, and provoke anxiety about the well-being and mortality of the patients.^32^ Consequently, health care teams must recognize the psychological support requirements of individuals and their family members at various stages of life with ADPKD. This involves engagement with support groups comprising social workers, psychologists, psychiatrists, and patient organizations dedicated to ADPKD.^33^ Another matter that should be taken into consideration is that social media content that is not based on evidence could also be a driver of psychosocial impact. Patients may experience frustration because of vague or insufficient information about various aspects of the disease, leading to negative emotions. There is a strong need for support in addressing the psychosocial needs of patients.^34^

Despite advances in understanding ADPKD, significant research gaps remain, particularly regarding its psychological aspects. Future investigations should explore the prevalence, causative factors, and effective management strategies for anxiety and depression within this population. Such research is vital for an approach to patient care in patients with ADPKD. Clinicians must recognize the unique challenges posed by ADPKD in managing QoL, including factors like kidney size, pain management, and psychosocial concerns. In addition, exploring how management strategies for ADPKD differ from those for other chronic diseases and whether psychosocial measures used in managing other kidney diseases are applicable to ADPKD could provide valuable insights for prompt interventions. Further studies are needed to develop precise assessment tools for QoL and to design interventions that specifically address the psychological needs of patients with ADPKD.
